# Feeding Habits in the Cultural Domains of Child Care: Elements for Health Promotion

**DOI:** 10.3389/fpubh.2021.536176

**Published:** 2021-03-03

**Authors:** Yolanda Martínez-López, Jaime Salvador-Moysén, Noé Alfaro-Alfaro

**Affiliations:** ^1^Academic Group of Public Health and Epidemiology, Institute of Scientific Research, Juárez University of the State of Durango, Durango, Mexico; ^2^Department of Public Health, University Center for Health Sciences, University of Guadalajara, Guadalajara, Mexico

**Keywords:** infant feeding, qualitative research, child care, health promotion, cultural domains

## Abstract

**Introduction:** Family eating behavior is determined by the meaning that the caretaker gives to food and the act of eating in the domestic environment, as well as the beliefs and perceptions around those concepts.

**Objective:** Identify the place that nutrition has within the dimensions of child care, the specific weight that the caregiver gives to it within the range of actions deployed and if there are differences when the child exhibits neurodevelopmental disorders, as a contribution to the design of interventions in health promotion.

**Methodology:** Qualitative, exploratory, two-stage study, with the approach of cognitive anthropology; proposal sampling of maximum differences, 121 informants participated in three groups, caregivers of: (1) healthy children, (2) children who had been hospitalized between 3 and 6 months prior to the time of the interview, and (3) children with a diagnosis of permanent neurological injury and that express some type of neurodevelopmental disorder.

**Results:** Nourishment is the element that reaches the highest values of cultural relevance in the three groups, is located in different domains according to the condition of the care receiver.

**Conclusion:** The common domains are Well-being, Health Maintenance, Coexistence, and Security, in the 3rd group the domain of Socialization emerges, the elements that make up the conceptual dimensions were identified, the comparative design allowed to identify differences. The description of the domains can represent the cognitive spaces of educational intervention and the elements that configure them are the triggers of the interaction, due to the importance they are given in everyday life.

## Introduction

The usual care that a young child receives includes actions that are aimed at guaranteeing survival, growth, and well-being. Nutrition, as an essential process for life, is a constitutive element of child care. There are multiple factors that intervene so that the diet that an infant receives meets her nutritional needs. Various studies have shown that socioeconomic conditions are strongly related to the ability to acquire food. It has also been observed that the existence of parental and social networks represent a support to mitigate precarious conditions and favor the nutritional conditions of groups with social and economic disadvantage (1, 2).

On the other hand, within the framework of the normative values of their environment, subjects generate cultural decisions and carry out actions in accordance with the information they have acquired and the way they have processed it. The meaning, beliefs and perceptions that the caregiver has of food and the act of eating in the family environment are related to the eating behavior of the family. It is important to highlight that from Anthropology common elements have been identified in the eating patterns, both of populations that inhabit different geographic contexts (3) and in others, with diverse historical antecedents, which is related to one of the most important characteristics that human beings have, their adaptive capacity to the natural and social conditions of the environment (4, 5).

The family environment is the scenario in which the caregiver decides the actions related to feeding, as a complex phenomenon, and thus, turns them into a stimulus and result of the integral child development process, in this way, in everyday life, she expresses, what Paris Aguilar mentions as “the capacity of the artisan production of facts and meanings” (6, 7).

Bronfenbrenner's theoretical approach (8, 9), referring to human development, is appropriate to understand the importance of food and the context in which it is carried out, both in its nutritional aspect and in its purpose of favoring the socialization of the child; processes carried out within the family and in which particularly the mother, constitutes an ideal mediating factor between the family nucleus and the school and social group. The close interaction with the mother in her immediate environment and the emotional attachment established between the mother-child dyad are elements that positively trigger the interconnection of the child's family microsystem with more complex environments, represented by the mesosystem, exosystem and macrosystem, structures social and cultural conditions that enable appropriate child development, and it is precisely the moment when the child is fed when the interaction, approach and communication of the mother-child dyad are closer.

In Mexico, food policies and programs have been designed that have allowed these highly complex structures, particularly health and education institutions, to interact favorably with the elements that are located in the immediate environment of school children and their mothers, with the purpose of maintaining health and promoting well-being. During the last 100 years, the actions of the food programs have been aimed at three fundamental aspects: (a) Support for food production, (b) Diversification and strengthening of the food collection, storage, and distribution network, and (c) Provision of subsidies and direct interventions to vulnerable groups to modify food consumption patterns, with a weak presence of educational interventions (10).

Despite the wealth of knowledge accumulated around the topic of infant nutrition, it contrasts with the difficult penetration observed in health promotion programs aimed at strengthening family eating practices, either to preserve favorable behaviors, or to modify or avoid negative behaviors. The approaches carried out about food and nutrition, both biomedical and epidemiological, which were pioneers in Mexico, were oriented more to the knowledge of environmental conditions, economic, and sociodemographic characteristics of individuals, than to the investigation of the aspects relevant social and cultural characteristics of the investigated communities (11, 12).

Although the relevance of these investigative approaches is indisputable, the information that would allow the design of health promotion programs was not used. In reality, the development of such programs has been based on the knowledge generated from the perspective of nutrition experts, that is, considering the dietary norm more than the social or cultural norm, so it is urgent to recover the look and testimony of those who care for and feed children, according to their beliefs and meanings (13, 14).

The interactions of social life are manifested in the small daily phenomena that progressively constitute the social fabric and that once all this has settled down can emerge a deeper knowledge closer to reality. This knowledge or familiarity is what Heidegger calls the world. The world is taken for granted and seen and is only noticed in situations of rupture; the worlds in which people live are not universal or timeless, on the contrary, they are different according to the culture, time or historical period and according to the family in which they are born. Qualitative distinctions give meaning to things recognized by the person in their daily life and are shaped by culture and language (15). Therefore, if health promotion programs are designed without taking into account the components of the cultural concept of food, without elucidating the reasons that underlie food preparation decisions, the health promotion programs will continue to advance in parallel lines promoting the health and behaviors of the population.

The model provided by the Cultural Consensus Theory explores how subjects generate cultural decisions and perform actions according to the information they have acquired and the way they have processed it, within their normative values. It allows this by recognizing the terms that the informants express as ideas or actions, the relevance that these terms have in the majority of the members of the group and the grouping pattern that is observed when looking for similarities between them, always from the point of view of the informants (16).

Weller and Romney define the cultural domain as a group of phrases or words linked by semantic relationships, which together refer to a specific conceptual sphere; they represent perceptions more than preferences; they derive their meaning, in part, from their position in a mutually interdependent system, which reflects the way in which a cultural group classifies the relevant conceptual sphere. It is possible to explore two aspects of the normative beliefs of the group: The qualitative aspect, through the grouping pattern of individual responses, while the quantitative aspect refers to the relevance attributed to each response as a result of the frequency of the response hierarchical.

There is consensus in the conceptual sphere when there is correspondence between the expressions of each informant with those of the rest of the group. Those informants who show greater correspondence with the group are classified as competent informants, due to the possibility that they contribute diverse and sufficient experiences as shared knowledge of the phenomenon under study.

To use the formal Cultural Consensus Model in the analysis of the information obtained by free lists and pile sort, it is necessary that the answers be words or short phrases, they are answered individually, they are oriented to the same topic with the same level of complexity, and respect the “fresh” answer, that is, without correction or transformation (17). The study of cultural domains has been used successfully before various health problems (18–20).

In order to identify the location of food within the cultural domains of child care, according to the health condition of the care recipient, a two-stage, phenomenological qualitative study was designed within the framework of the cultural consensus theory.

### General Objective

Identify the site and the relevance given to food within the cultural dimensions of child care.

### Specific Objective

Know the differences in the configuration of cultural domains when the care recipient suffers temporary or permanent illness.

### Design

Qualitative exploratory study, in resident population of the city of Durango, Dgo., Mexico.

#### Informants

Three groups of preschool child caregivers were formed by propositive sampling and maximum differences:

Group 1−38 healthy child caregivers,

Group 2−40 caregivers of children who had been hospitalized between 3 and 6 months prior to the time of the interview, and

Group 3−43 caregivers of children diagnosed with permanent neurological injury and who expressed some type of neurodevelopmental disorder.

In determining the number of participants per group, the assumption of the consensus model was taken as the main criterion, which suggests for studies of cultural description a minimum size of 17 informants to classify 95% of the questions correctly, because the correlation Average among informants tends to be high. The participants of each group were divided equally for the realization of the two stages of the work.

### Operational Stages

The meeting scenarios for the collection of information were the pediatric service of the General Hospital of Durango, Dgo and the Integral Development Care Center “Dr. Isauro Venzor (CADI) of Durango. One hundred and twenty one semi-structured interviews were conducted, fifty-eight and sixty-three respectively, free listings were obtained. The response rate was 100%.

The information was analyzed with support of Anthropac 4.1 and Ucinet software.

Stage 1. Obtaining free listings. They were prepared by registering the first 10 short sentences or words that the informant mentioned when asked what she was looking for child care; once the free listings were collected and in a tabular form the particular elements were identified, the absolute and relative frequency with which each of these elements is mentioned and the place in which they appeared in the lists, the relevance index was determined cultural or smith index, which is the product of the relationship between the previous values, that score represents the specific weight that each element has as part of the concept of care, however, it does not allow to discover the relationship that each of these elements have with each other, nor the way in which are configured the cultural dimensions, for that the next stage is necessary.
Stage 2. Drawing lots. The 20 elements that reached the highest figures of the cultural relevance index (smith index) were selected and printed on cards; the cards were assigned the numerical code corresponding to the cultural relevance index, so that the components of each group could be identified and individual values assigned. The participants gathered the cards that had the terms that looked the most and formed 4–5 lots each.

Individual values were analyzed using a factorial model that estimates the likelihood that a homogeneous system of knowledge predominates in a culture, based on the correspondence between the knowledge of the group and those of the individual. The likelihood criterion for obtaining consensus in the models implies that the first factor reaches a ratio three times greater than that of the second factor, condition that is interpreted as concordance in the responses of the informants, it is also possible to identify the similarity and the difference between the dimensions that make up the cultural domains of each group (21, 22). The aggregate values were analyzed by hierarchical conglomerates, to identify the degree of perceived similarity among the participants and is represented in a tree diagram with correlation levels.

## Results

The primary caregiver is the mother in the caregiver groups of the three groups of children, although the participation of the father is more frequent in the group of children with developmental disorders than in the other two groups of children (Table 1). Nourishment is the element that reaches the highest values of cultural relevance in the three groups of caregivers, it is appreciated that it occupies the first and second place among the first 20 elements (Table 2).

**Table 1:** Characteristics of the participating groups.

	**No. of participants**	**Child age (mean)**	**Caregivers age (mean)**	**Reason by gender male/female**
**Group A**				
Healthy Child Caregivers	38	24 months	27 years	1/11.6
**Group B**				
Caregivers of hospitalized children in the past three to six months	40	24 months	28 years	1/9
**Group C**				
Caregivers of children with permanent neurological injury	43	37 months	37 years	1/7.6
**Total**	121			

**Table 2:** Elements of greater cultural relevance, conformation of the domains by pile sort.

**Cultural relevance**		**Domain**	**Cultural relevance**		**Domain**	**Cultural relevance**		**Domain**
1	Feeding	2	1	Feeding	1	1	Personal care	2
2	Avoid accidents	5	2	Homeclean	3	2	Feeding	4
3	Take care	3	3	Child cleaning	3	3	Cleaning	2
4	Loving	3	4	Watch out	2	4	Avoid accidents	3
5	Homeclean	2	5	Avoid accidents	1	5	Be aware	2
6	To be alert	2	6	Watch what he eats	1	6	Hygiene	2
7	Watch over	5	7	To play	2	7	Give love	4
8	Cleanliness	2	8	Clean toys	3	8	Don't approach fire	3
9	Bath	2	9	Coexistence	2	9	Correct behavior	4
10	To play	3	10	Attention	2	10	Family life	4
11	Coexistence	5	11	Loving mother	2	11	Attend needs	1
12	Education	2	12	Give medicine	1	12	Concer	1
13	Avoid disease	1	13	Go to the doctor	1	13	No things to mouth	3
14	Therapy	4	14	Communication	2	14	To play	4
15	Go to the doctor	1	15	Necessary medicine	1	15	Watch out	2
16	Give medicine	1	16	Hygiene	3	16	Fatigue	1
17	Stimulation	4	17	Enough sleep	1	17	Go to the therapy	1
18	Follow	4	18	Patience	2	18	Don't approach plugs	3
19	Patience	3	19	Love	2	19	Don't leave home	3
20	Deceive	5	20	No pets	3	20	Education	4

The conceptual dimensions of “child care” show homogeneity, the percentage of accumulated variance for the first factor is 81.3, 84.6, and 86.0, respectively, when it is >60%, it is homogeneous—and consensus is observed in the three groups, the ratio between the first and second factor is 3.67, 4.03, and 4.53—when it is >3, consensus is considered to exist (Table 3).

**Table 3:** Cultural relevance of food and Consensus of cultural domains of “child care,” according to group of caregivers.

	**Cultural relevance (CR)**		**Cultural consensus**		
**Group**	**Place of the feeding between the 20 elements**	**Value of CR**	Factor 1 (F1)^*^ and % of cumulative variance	**Factor 2 (F2) and % of cumulative variance**	Ratio F1/F2^**^
1 *n* = 38	First	0.238	6.709	1.827	
			81.3%	93.4%	3.67
2 *n* = 40	First	0.355	8.207	2.034	
			84.6%	94.9%	4.03
3 *n* = 43	Second	0.200	9.153	2.018	
			86.0%	95.3%	4.53

*Durango, Dgo., Mexico*.

* *Cumulative variance of F1 >60% = It is homogeneous*.

** *Ratio F1/F2 > 3 = There is consensus*.

The dimensions that appear in the three groups show similarities because of the presence of the particular elements, however, when observing the arrangement and the relationship between them, the dimensions are configured differently.

**Figure 1:**
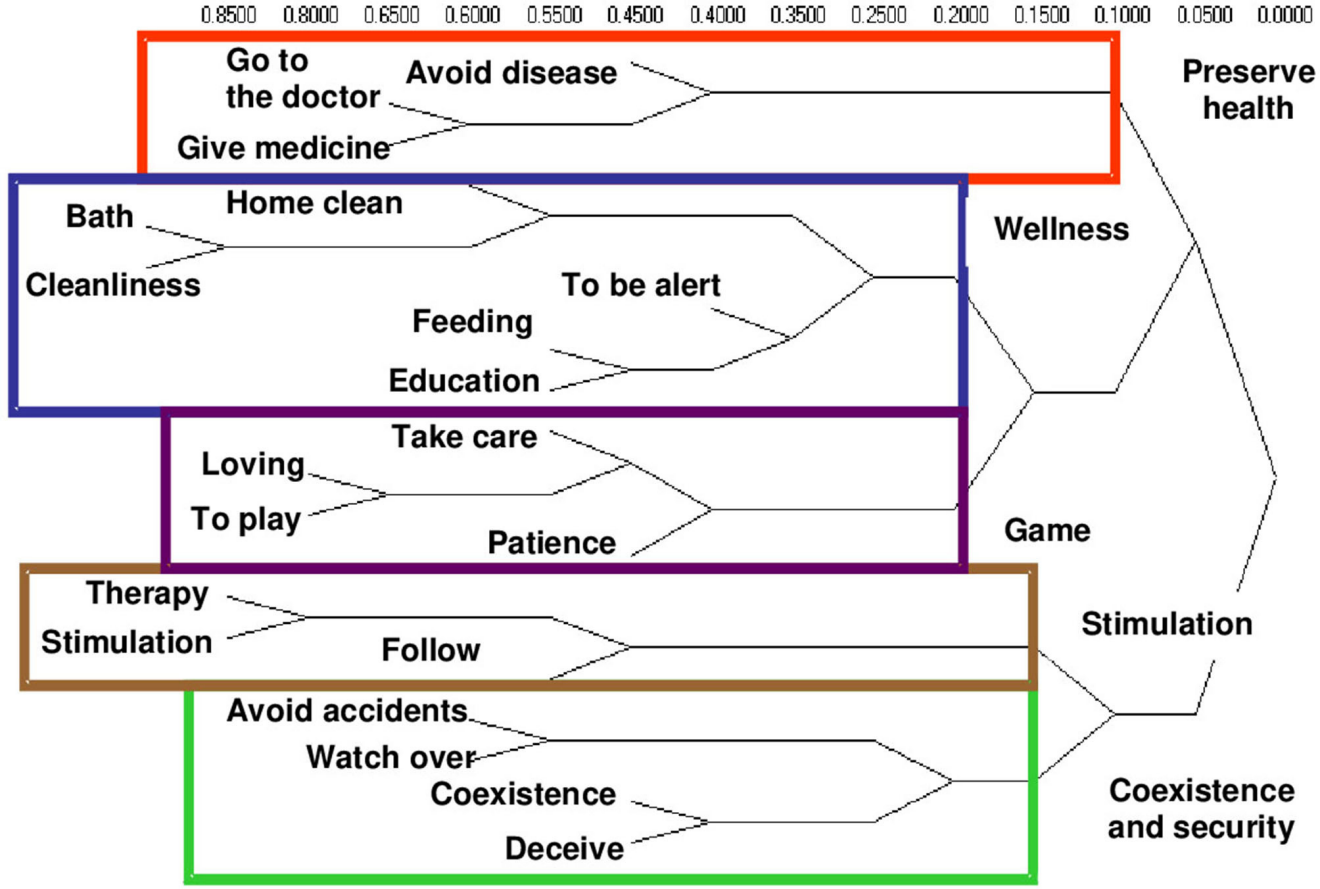
Conceptual dimensions of child care by hierarchical conglomerates. Group 1. Caregivers of healthy children.

In group 1, made up of the mothers of healthy children, food is part of the welfare dimension, which includes body hygiene, bathing and household cleaning, among others (Figure 1).

**Figure 2:**
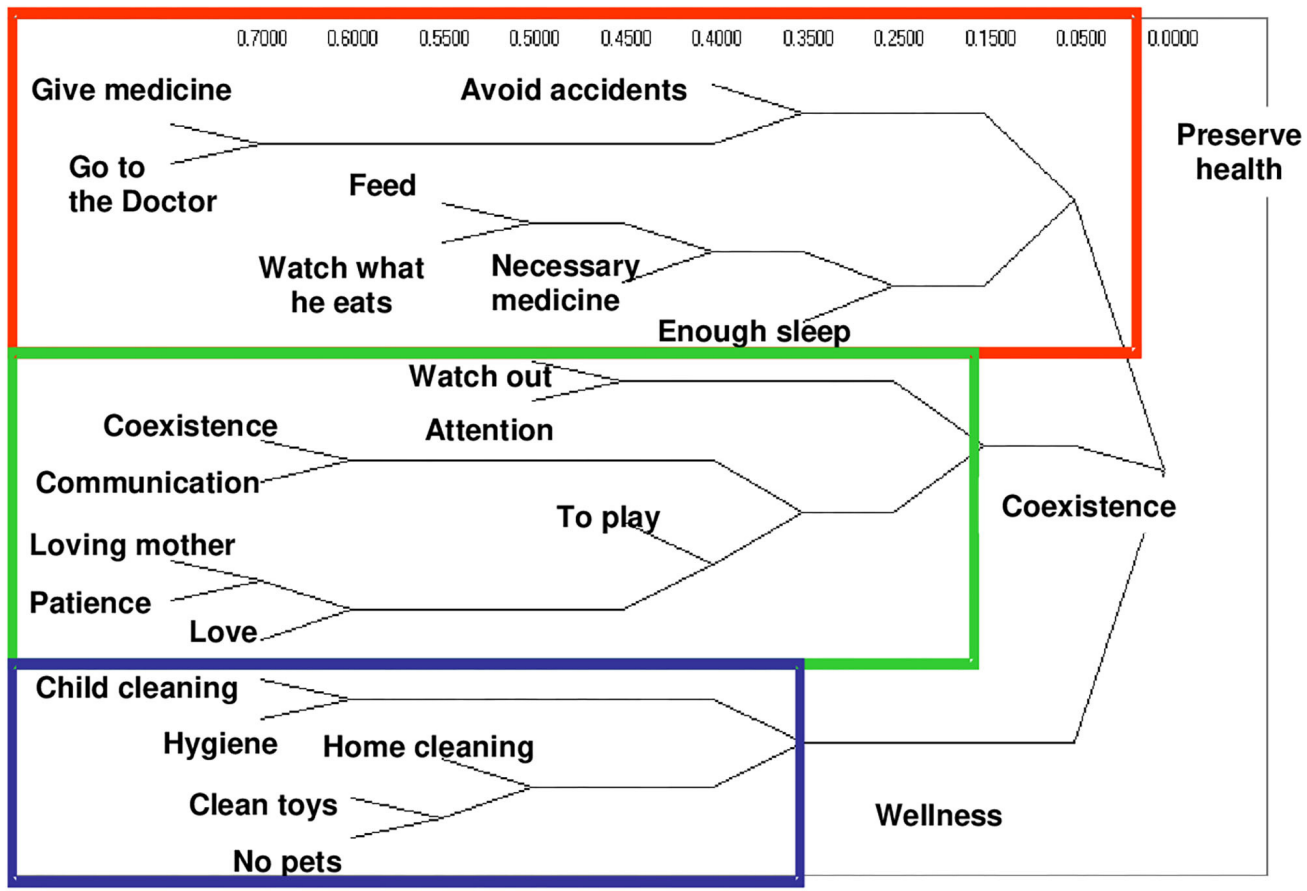
Conceptual dimensions of child care by hierarchical conglomerates. Group 2. Caregivers of children hospitalized 6 months ago.

**Figure 3:**
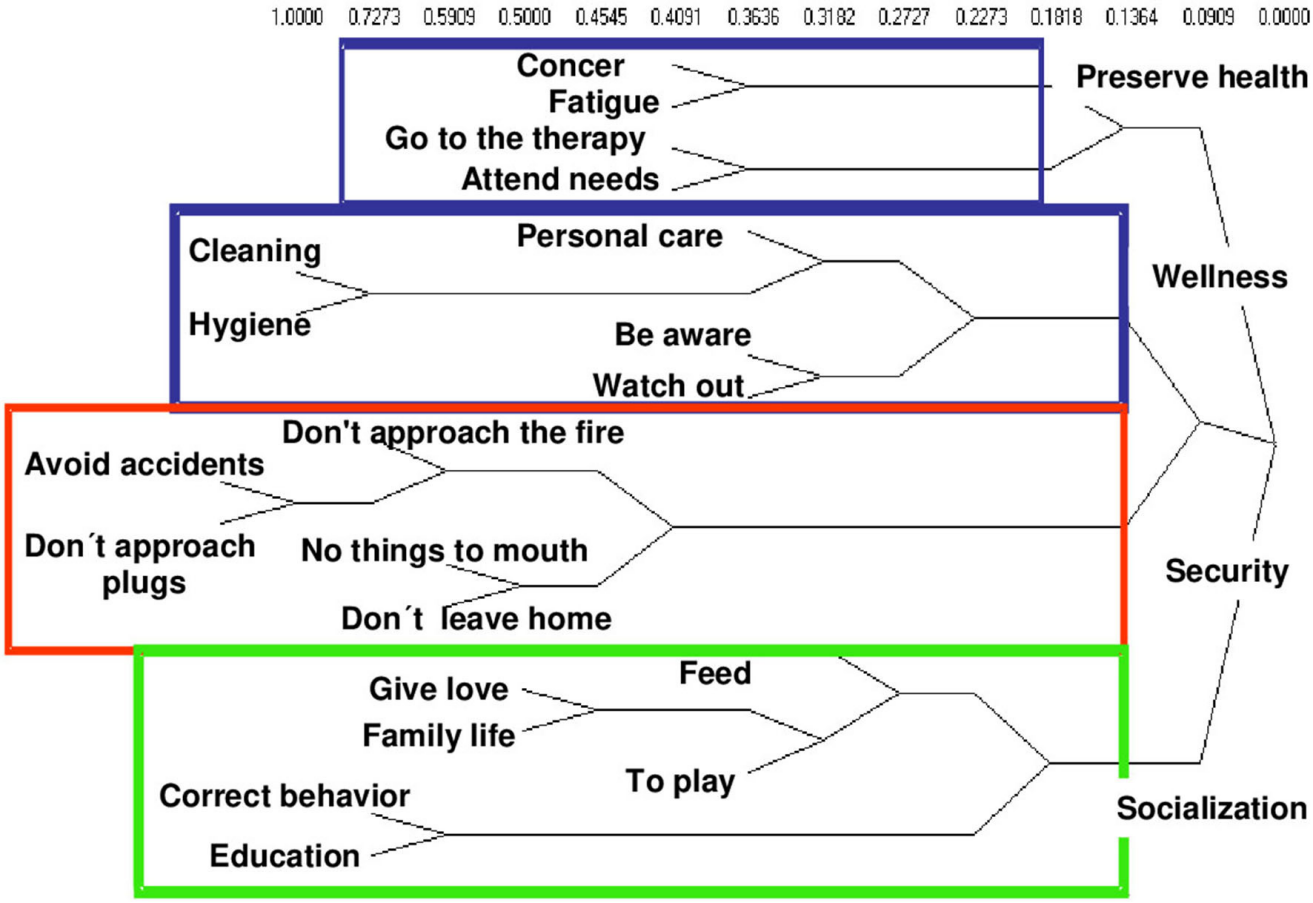
Conceptual dimensions of child care by hierarchical conglomerates. Group 3. Caregivers of children with developmental disorders.

In group 2, that of the caregivers of children who have had a period of hospitalization between 3 and 6 months prior to the time of the interview, feeding is located in the dimension of health conservation what is accompanied by the care in what they eat and also in buying the necessary medicines (Figure 2). While in group 3, caregivers of children with neurodevelopmental disorders place food in the socialization dimension, in which they share space with actions aimed at avoiding social isolation, and favors the incorporation of child to the family environment, the neighborhood, and the school (Figure 3).

## Discussion

The use of an anthropological approach in the study of the feeding of preschool children with different health conditions, allows to have an appropriate cultural and social perspective, to understand the impact that the caregivers of these children have, in the care they grant on the nutritional and relational level, particularly when it comes to preschoolers with neurological damage.

Infant feeding is the field in which the mother performs one of the activities that represents a relevant dimension of parenting and that she perceives as a significant feature of “being a mother,” which involves participating and deciding on the child's development. It is important to mention that it is also a space for participation and decision of the baby about its immediate environment. It is a time of intense interaction where the needs of the baby and the care of the mother are faced.

The emotional symbolic universes make possible the cultural patterns and norms that modulate both the form of language acquisition and the behavior patterns that allow communication, the exchange between the subjects, and the reproduction and transformation of the social order (23).

The group of caregivers of children with permanent injuries identify feeding on the same level as “play, give love, family life, correct behavior, and education,” so that a nuance of socialization is appreciated; unlike what the other groups refer to, this is part of the dimension in which recreational, affective activities that promote discipline and education are incorporated; they give great cultural relevance to alimentation for different reasons than the caregivers of the other two groups, these caregivers have more experience in the home care process, they seem to have more complex cultural constructions on this phenomenon, they have turned feeding into a space for interaction, they consider it as the opportunity to modify or adapt the behaviors linked to maternage, particularly related to the conditions of the baby's social performance, of the mother and the rest of the family, allows them to identify problems in the relationship established by the child with those responsible for their care, it could be that they have left behind hospital crises, family crises and the grief of not having had a healthy child; the caregivers are located in another plane, which becomes more understandable from the theoretical perspective of Bronfenbrenner and Morris (24), who defines human development as an enduring change in the way in which the person perceives the environment and relates to it. Although the Bronfenbrenner model has the child as the central protagonist, the importance of the caregiver's role is unquestionable, as a character that is responsible for “creating” the environments and “building” relationships, which allow the child to be optimally incorporated into its environment; if this is important when it comes to a healthy child, in the case of children with impaired development, it is crucial. To understand the feeding habits from a cultural perspective, it is necessary to design methodological approaches that allow access to symbolic universes and the study of social processes that are articulated with their properties and meanings, particularly the internal meanings of alimentation (3).

## Conclusion

The elements that make up the conceptual dimensions of child care were identified, as well as the prominent role given to food. The comparative design allowed to identify substantive differences in the perception that the caregiver has about what feeding means. When the child is healthy, she is fed to maintain well-being. If the child becomes ill, food becomes a necessary element to regain health and avoid illness, while when the child is faced with a developmental alteration as a permanent condition, the physical and temporary space in which the food, is used to promote social interaction. The meaning that the caregiver gives to food can be the starting point in the design of health promotion programs.

If the caregiver received guidance that allowed her to reinforce the perception of the usefulness of the diet, a bond of trust would be created with the health personnel, due to the consonance of the approaches and from there, the reception of the messages could be facilitated and practices aimed at establishing healthy eating habits.

## Data Availability Statement

All datasets generated for this study are included in the article/supplementary material.

## Ethics Statement

The studies involving human participants were reviewed and approved by this work is part of a Doctoral Thesis. The ethical aspects of the project were reviewed and approved by the Doctoral Program Tutorial Committee of the University of Guadalajara, Mexico. Participating mothers were informed and invited to participate in the study. They expressed their acceptance and signed an informed consent letter. The research consisted of conducting interviews with the participating mothers. The questionnaires are anonymous and confidential. Written informed consent to participate in this study was provided by the participants' legal guardian/next of kin.

## Author Contributions

The contributions of YM-L, JS-M, and NA-A were similar in theoretical, method, and results analysis aspects. YM-L wrote the article. All authors contributed to the revision of the manuscript, read it, discussed, and approved the final version.

## Conflict of Interest

The authors declare that the research was conducted in the absence of any commercial or financial relationships that could be construed as a potential conflict of interest.
